# Effects of cyclosporin, nifedipine and phenytoin on gingival myofibroblast transdifferentiation in monkeys

**DOI:** 10.1590/1678-7757-2018-0135

**Published:** 2018-11-08

**Authors:** Claudia Misue Kanno, Jose Americo de Oliveira, Edilson Ervolino, Ana Maria Pires Soubhia

**Affiliations:** 1 Univ. Estadual Paulista, Faculdade de Odontologia, Departamento de Emergência, Araçatuba, São Paulo, Brasil; 2 Univ. Estadual Paulista, Faculdade de Odontologia, Departamento de Ciências Básicas, Araçatuba, São Paulo, Brasil; 3 Univ. Estadual Paulista, Faculdade de Odontologia, Departamento de Patologia e Propedêutica Clínica, Araçatuba, São Paulo, Brasil

**Keywords:** Gingival overgrowth, Cyclosporine, Phenytoin, Nifedipine, Myofibroblast

## Abstract

**Objective:**

Myofibroblasts have been associated with the development of several pathologic fibrotic conditions. This longitudinal study aims to assess the proliferative and antiapoptotic effects of cyclosporin, nifedipine and phenytoin on gingival connective tissue cells of nonhuman primate, as well as to analyze a possible role of myofibroblasts in gingival overgrowth.

**Materials and Methods:**

Gingival samples from the right superior canine area were obtained from 12 male monkeys ( *Sapajus spp* ) to comprise the control group. After one week, the animals were randomly assigned to three groups, which received daily oral doses of cyclosporin, nifedipine or phenytoin for 120 days. Gingival samples were collected from the left superior canine area of two animals of each group at 52 and 120 days. Histological sections were stained with hematoxylin and eosin, and immunoreacted against α-SMA, Ki- 67 and bcl-2.

**Results:**

α-SMA immunoreaction was negative in the control and experimental groups. Similarly, no difference between groups concerning immunostaining against Ki-67 and bcl-2 was observed in connective tissue cells.

**Conclusion:**

Based on this methodology, it may be concluded that gingival overgrowths induced by cyclosporin, nifedipine and phenytoin are not associated with neither myofibroblast transdifferentiation, proliferation nor apoptosis of gingival connective cells in monkeys.

## Introduction

Drug-induced gingival overgrowth (GO) is an unwanted side effect that has been mainly associated with chronic therapy with phenytoin, nifedipine and cyclosporin (CsA). This condition is characterized by gingival enlargement due to alterations in cellular and extracellular matrix (ECM) metabolisms, leading to accumulation of several components of ECM. ^1^ ^–^ ^2^ However, the inducing molecular mechanisms remain unknown in many aspects.

The development of GO involves changes in the metabolism of several cellular types, in a complex interdependent reaction in cascade. ^2^ ^–^ ^4^ However, fibroblasts have been the main cell studied, as they are responsible for the synthesis and enzymatic degradation of ECM proteins. ^3^ ^,^ ^5^ ^–^ ^9^ Fibroblasts derived from phenytoin-induced GO exhibit higher proliferative activity ^10^ and elevated levels of collagen synthesis ^11^ compared with cells obtained from normal gingiva. This fact raised the hypothesis that phenytoin exerts a selective pressure favoring the proliferation of a specific fibroblastic subpopulation, which eventually becomes the predominant cell type in the tissue. Ultrastructural analysis of "modified" fibroblasts of GO induced by phenytoin showed morphological features compatible with myofibroblasts. ^12^

Myofibroblast is considered the main cellular type involved in extracellular matrix deposition during tissue remodeling. This cell population is characterized by the expression of α-smooth muscle actin (α-SMA). After the completion of physiological events, such as wound healing, the reversion of myofibroblasts does not seem to be possible, and massive apoptosis has been described. ^13^ Myofibroblast apoptosis is critical for normal healing as its persistence favors a continuous ECM deposition and the development of pathologic fibrotic conditions. ^14^

CsA, phenytoin and nifedipine induce fibrosis not only in gingival tissue, but also in important organs that may present a resultant loss of function. Studies in rats showed increased levels of α-SMA immunostaining in kidneys ^15^ ^–^ ^16^ and pancreas ^17^ after therapy with CsA. Additionally, Dill & Iacopino ^12^ (1997) described cells with ultrastructural features of myofibroblasts in liver, spleen and mesentery of rats, after phenytoin administration. These facts raise the question of whether CsA, phenytoin and nifedipine might induce myofibroblast transdifferentiation and proliferation in GO.

GO is a multifactorial lesion that involves complex and interdependent interactions among different cell types. ^3^ ^–^ ^4^ For this reason, important new insights may be gained into GO pathogenesis with *in vivo* studies, employing standardized time of analysis. Therefore, the aim of this study was to analyze the effects of CsA, nifedipine and phenytoin on myofibroblast transdifferentiation, proliferation and resistance to apoptosis in gingival tissue of tufted capuchin monkeys ( *Sapajus spp* ).

## Material and methods

The experimental protocol of this study was approved by the Committee of Ethics in Animal Experiment (Protocol 41/03). This study is part of a series of researches carried out on the same material and followed the Guide for the Care and Use of Laboratory Animals.

Twelve healthy male tufted capuchin monkeys ( *Sapajus spp* ) were used in this study. This animal model was used for its anatomical similarity and phylogenetic proximity to human. They were maintained in individual steel cages, with natural light and fresh air during the day and under artificial warming during cold nights. Their diet was based on fruits, vegetables, yogurt, eggs and water *ad libitum.*

Before surgery, the animals were sedated and anesthetized with intraperitoneal injection of sodium thionembutal (Abbot Laboratories, Chicago, IL, USA) at 30 mg/kg body weight. Surgical sites were classified according to the criteria for Sulcus Bleeding Index by Mühlemann and Mazor ^18^ (1958) and anesthetized with routine dental infiltration anesthesia with 2% mepivacaine with 1:100,000 adrenaline (Mepiadre^®^, DFL, Rio de Janeiro, RJ, Brazil). Gingival samples, comprised of free and attached gingiva, were obtained from the right superior canine area of all animals, which constituted the control group. The vertical incisions included the mesial and distal interdental papillae on the buccal side and were completed with an apical incision at the mucogingival junction.

The animals were randomly assigned to three experimental groups one week after the control biopsy (n=4). Group I was treated with daily oral doses of 5 mg/kg of CsA (Novartis Pharma AG, Basle, Switzerland) whereas Group II received 7.5 mg/kg of phenytoin (Apothicario, Araçatuba, SP, Brazil). One week after beginning the drug administration, the dosage of both medications was increased to 15 mg/kg. The gradual increase in drug dosage allowed observation of possible toxic effects as no previous study in drug safety in this animal model was found, especially regarding phenytoin. The determination of final CsA dose was based on the recommended human posttransplant dosage. Group III received daily oral doses of 40 mg/kg of nifedipine (Apothicario, Araçatuba, SP, Brazil) during the whole experimental period. The drugs were diluted in yogurts and provided once a day.

Gingival specimens were obtained from the left superior canine area of two animals of each group on the 52^nd^ day. The experimental period was concluded on the 120^th^ day, when samples were obtained from the left superior canine area of all animals not subjected to biopsy on the 52^nd^ day. Sampling was performed at the opposite side of the first biopsy to avoid the interference of wound healing. The biopsy on the 52^nd^ and 120^th^ days followed the same protocol of the first sampling procedure, except for the use of ketamine (Cristalia Produtos Químicos Farmacêuticos Ltda, Itapira, SP, Brazil) at 10 mg/kg, as general anesthetic.

The animals were observed during the whole experimental period in the search for possible adverse effects of drug therapy. Clinical images were registered on the three days of tissue sampling.

### Immunohistochemical study

Gingival samples were fixed in 10% formalin solution, paraffin embedded and sectioned in buccolingual plane, following the tooth long axis. 6-μm-thick sections were stained with hematoxylin and eosin for morphologic analyses of epithelial and connective tissues.

For immunohistochemical study, 3μm-thick sections were deparaffinized and subjected to epitope retrieval in steamer. For α-SMA study, the slides were maintained in citrate buffer at 60°C for 20 minutes. Tris-EDTA at initial temperature of 60°C and then heated to 95°C for 20 minutes was used as buffer solution before the proliferation marker, Ki-67 primary antibody, and bcl-2 immunoreactions for the assessment of resistance to apoptosis. Overnight incubation was carried out at 4°C with the following dilutions of primary antibodies: Monoclonal mouse anti-human α-SMA (M0851) 1:200 (Dako Corp., Carpenteria, CA, USA), Monoclonal mouse antihuman bcl-2 (M0887) 1:50 (Dako Corp., Carpenteria, CA, USA) and Monoclonal mouse anti-human Ki-67 (M7240) 1:100 (Dako Corp., Carpenteria, CA, USA). Sections were washed in tris-buffered saline and incubated with streptavidin complex with biotinylated horseradish peroxidase (Dako Corp., Carpenteria, CA, USA) for 30 minutes, then diaminobenzidine (Dako Corp., Carpenteria, CA, USA) was used as a chromogen.

Fast green dye (Dako Corp., Carpenteria, CA, USA) was used as a counterstain, except for samples reacted against bcl-2 in which hematoxylin was used. As negative control, gingival sections from tufted capuchin monkeys were subjected to the same immunohistochemical reaction protocol, without the primary antibody. Positive control reactions for bcl-2 and Ki-67 were carried out in spleen and/or tonsils of monkeys of the same species. Immunoreaction in blood vessels was considered a positive internal control for α-SMA.

Histological images were captured for analysis in a computer program for image processing (Leica Microsystems, Heerbrugg, Switzerland). Two representative sections of each animal relative to the periods under study were analyzed at 200x magnification. Fibroblast-like cells were counted in HE sections in three different connective tissue sites, with corresponding areas of 600.000 μm ^2^: subjacent to oral epithelium (OE), sulcular epithelium (SE) and middle-deep area (MD). Cell counting was blinded performed in two different occasions by two independent examiners. Myofibroblast cell counting followed the same protocol.

## Results

### Clinical observations

The experimental period elapsed without any intercurrence. No clinical or behavioral modification that could be attributed to drug administration or its systemic side effects was observed.

At the initial phase (control group), subtle signs of gingival inflammation were observed, usually limited to the free gingival margins.

**Figure 1:**
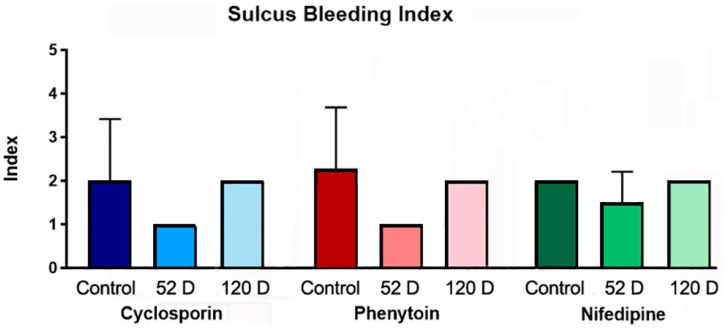
Sulcus Bleeding Index at days 0 (Control), 52 and 120. Medium values of three points of measurement at the buccal side of superior canines

Gingival enlargement was observed in all animals, in a variety of severity grades and more prominent in anterior areas. CsA induced the most prominent enlargement, observable from the second week of drug administration. No significant difference between control and treated groups was found with regard the Sulcus Bleeding Index ( Figure 1 ), although a tendency for lower values was observed in some control groups.

### Histomorphological study

Control transversal sections disclosed clearly distinctive layers of connective tissue. The papillary layer of lamina propria was consisted of loose connective tissue with a profuse capillary network. Deeper to this layer, an extensive area of dense irregular connective tissue, with thick bundles of collagen fibers was observed. This reticular layer presented larger blood vessels enclosed in isolated areas of loose connective tissue, centrally and deeply situated. In the area of junctional epithelium, the underlying connective tissue frequently showed varied inflammatory infiltrate grades.

**Figure 2:**
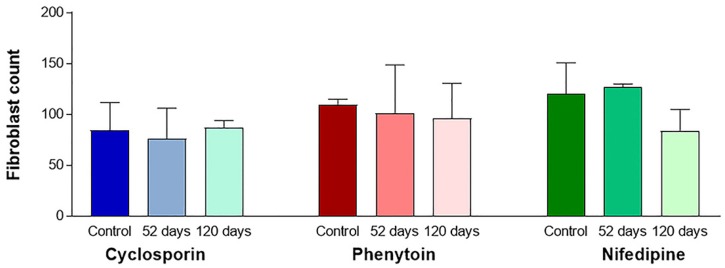
Fibroblast-like cell counting at days 0 (Control), 52 and 120. Medium values of three areas with 600.000 mm ^2^: subjacent to oral epithelium, sulcular epithelium and middle-deep area

The histological aspects were maintained on 52- day samples of all experimental groups, except for the visibly enlarged extension of the sections at the macroscopic level. Occasionally, the lamina propria underlying the oral epithelium presented a longer and larger papillae, despite the drug administrated. This histological feature was more prominent on the 120- day samples of all the experimental groups. Statistical analysis did not disclose differences in mean values of fibroblast-like cell counting between periods in the three experimental groups ( Figure 2 ).

### Immunohistochemical analysis

**Figure 3:**
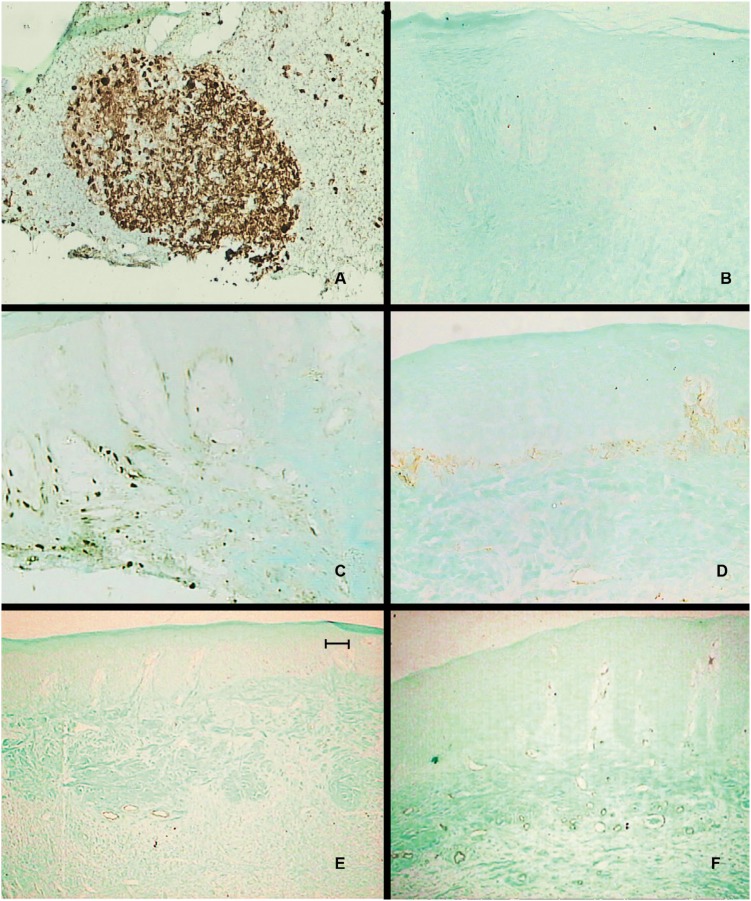
Ki-67 and α-SMA immunostaining. (A) Ki-67 immunostaining in positive control in histological section of lymph node of capuchin monkey ( *Sapajus spp* ). Original Magnification x20. (B) Negative control in histological section of gingiva of capuchin monkey. Fast green dye counterstaining. (C) Immunoreaction against Ki-67 in buccolingual transversal section of gingival sample of the control group. Fast green dye counterstaining. (D) Immunoreaction against Ki-67 in buccolingual transversal section of gingival sample of phenytoin group, 120 days. Fast green dye counterstaining. (E) Immunostaining against α-SMA in gingival sample of the control group. Fast green dye counterstaining. Original Magnification x10. (Bar=50 μm) (F) Immunoreaction against α-SMA in gingival sample of the CyA group (52 days). Positive reaction in blood vessels. Fast green dye counterstaining

Ki-67 immunostaining was detected in a scattered and non-uniform pattern in the nucleus of basal epithelial cells of some samples of the control group. Conversely, this immunostaining was constant and profuse in blood vessel cells, especially in the papillary layer ( Figure 3C ). The same pattern of epithelial immunostaining was infrequently observed in the treated groups, and even more limited in the connective tissue ( Figure 3D ). No reaction was detected in the negative control samples.

α-SMA immunostaining was restricted to the cytoplasm of endothelial cells and was more evident in medium-diameter arteries and veins. These vessels had a profuse distribution in the connective tissue of the papillary and reticular layers of lamina propria. No additional labeling was detected in any other cell type, neither in the control ( Figure 3E ) nor in the treated samples in the three experimental periods ( Figure 3F ). The absence of myofibroblasts precluded statistical analysis.

**Figure 4:**
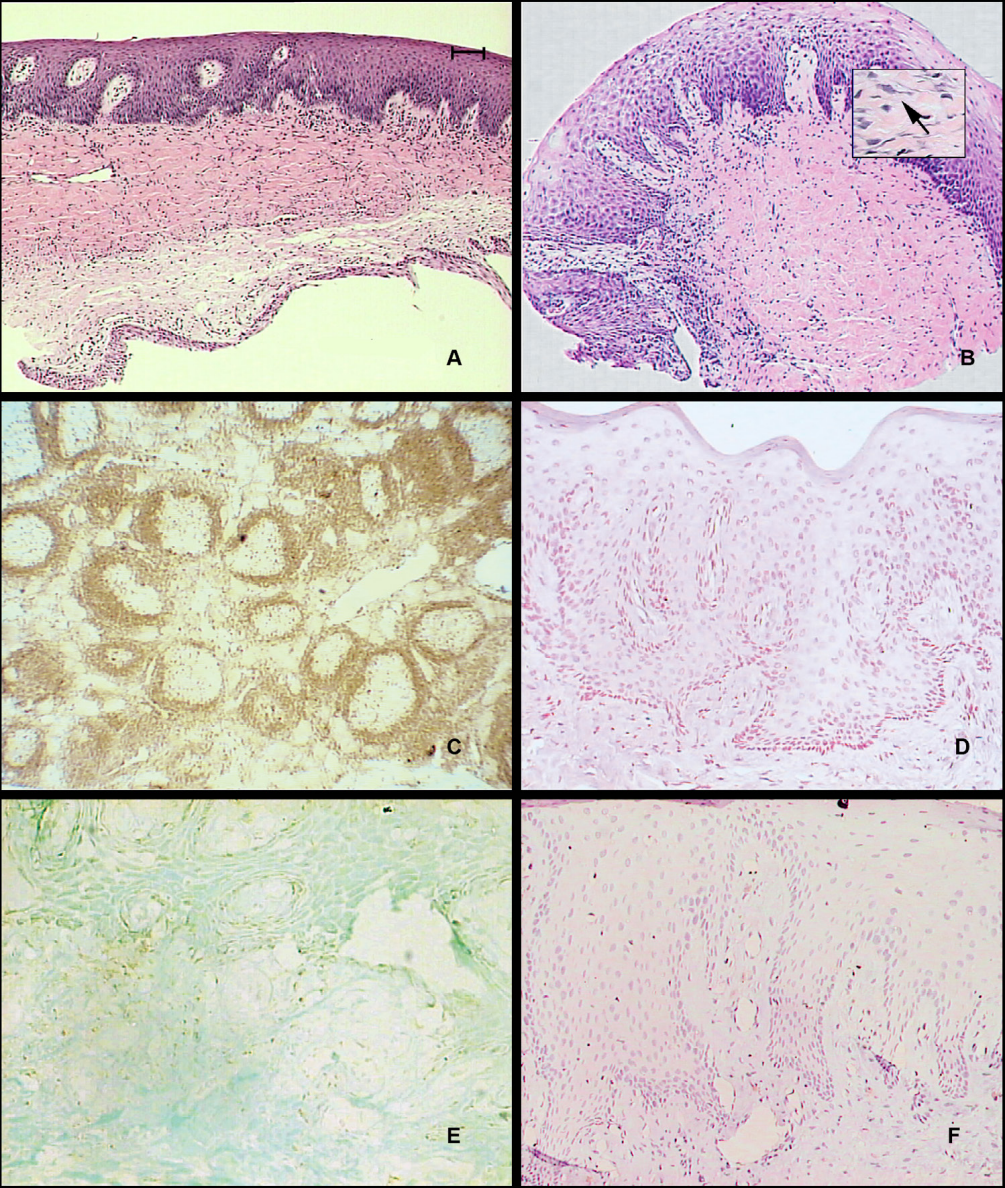
Photomicrographs of hematoxylin and eosin and bcl-2 immunostaining. (A) Gingival sample of the control group. HE. (Bar=50 μm). (B) Gingival sample of the phenytoin group, 120 days. In detail, fibroblast-like cell (arrow). (C) Positive control of bcl-2 immunoreaction in histological section of spleen of capuchin monkey ( *Sapajus spp* ). Original Magnification x10. (D) Negative control in histological section of gingiva of capuchin monkey. Hematoxylin counterstaining. Original Magnification x20. (E) Immunoreaction against bcl-2 in gingival sample of the control group. Fast green dye counterstaining. (F) Immunoreaction against bcl-2 in gingival sample of the CyA group, 120 days. Hematoxylin counterstaining

A sparse distribution of bcl-2 immunostaining was observed in the cytoplasm of epithelial cells of the control samples ( Figure 4E ). No immunostaining was detected in this area in samples of the treated groups, in any experimental period ( Figure 4F ). In some sections, the reaction against bcl-2 was observed in non-uniform pattern in different layers of the epithelium relative to the sulcular area and its underlying connective tissue, supposedly in inflammatory cells. This detection varied according to the severity of inflammatory infiltration in this area in different animals and it was not correlated with any drug administered. The positive control consisted of immunoreactions in the germinal center of spleen and lymph nodes. No reaction was detected in negative controls.

## Discussion

To the best of our knowledge, this study marks the first time that a possible myofibroblast participation in the development of GO induced by nifedipine, phenytoin or CsA is longitudinally assessed in a nonhuman primate.

*In vitro* studies with gingival fibroblasts described an increase in α-SMA expression induced by CsA, associated with the upregulation of TGF-β1 gene expression. ^19^ This cytokine is a potent inducing factor for myofibroblast transdifferentiation ^13^ and protects them from apoptosis. ^14^ Parallelly, *in vivo* studies found a correlation between the fibrosis induced by CsA in kidneys ^15^ ^,^ ^16^ and pancreas of rats ^17^ with an increase in α-SMA immunostaining. Then, the association between myofibroblast proliferation and GO seemed to be plausible. However, this hypothesis could not be proven true in this assay, similarly to a previous *in vivo* study with CsA in rats. ^20^

The molecular pathways of myofibroblast differentiation involve a combined set of cytokines, growth factors, ECM composition and mechanic tension, molecules on cellular surface that, among other factors, ^15^ may not be associated with GO. Drug effects vary according to the cell type. Myofibroblasts are, presumably, originated from preexisting fibroblasts, perivascular fibroblasts and pericytes, ^21^ endothelial cells, ^22^ stem cells from bone marrow ^23^ and epithelial cells. ^16^ It is not clear whether different origins of myofibroblasts are correlated with different biological events, such as normal tissue repair and pathological fibrosis, ^15^ or even, whether myofibroblasts in different organs are derived from specific cellular sources. Moreover, the resultant tissue response is still a consequence of a complex interaction between local microenvironment conditions, a set of paracrine factors provided by specific neighboring cells, ECM composition and integrins. ^15^

Signaling pathways for myofibroblast transdifferentiation induced by CyA are active in gingival fibroblasts. ^19^ However, they seem to be expressed in lower levels by gingival fibroblasts maintained in three-dimensional culture, ^24^ which indicates an inherent and genetically determined feature of this fibroblast phenotype. ^25^ Therefore, increased rates of TGF-β1 induced by CyA ^19^ as well as nifedipine and phenytoin, ^26^ do not seem to be sufficient, by themselves, for the induction to *in vivo* myofibroblast trandifferentiation in GO. ^20^ Anyway, elevated TGF-β1 levels are also characteristic of inflammatory processes, although the increase in this growth factor levels in GO cannot be explained solely by gingival inflammation ^10^ . Despite the controversies over the role of inflammation on GO pathogenesis, it tends to be consider an important contributing factor but not essential for the development of GOs. ^27^ Interestingly, myofibroblast proliferation by itself could not lead to fibrosis induced by CsA in pancreas of rats, and an inflammatory component was determinant for the increase in ECM components. ^17^ In this study, no correlation was found between GO development and the Sulcus Bleeding Index.

Fibroblast proliferation induced by CsA is directly proportional to the severity of inflammation as a decrease in immunostaining against Ki-67 occurs after the control of periodontal irritants. ^28^ Consequently, an equivalent cellularity can be observed when normal gingiva and CsA-induced GO are compared. ^29^ In this study, the restricted and low-intensity inflammation observed histologically may justify, at least partially, the limited immunostaining against Ki-67 in gingival connective tissue in all experimental groups, which is in accordance with the results of fibroblast-like cell counting.

The absence of myofibroblasts is compatible with the concept that GO develops mainly at the expense of a decrease in ECM degradation, more than the synthesis of its components. In this sense, decrease in collagenolytic activity induced by CsA ^3^ ^,^ ^7^ and phenytoin ^9^ ^,^ ^30^ were described in the literature. Additionally, previous studies described a decrease in collagen amount or its gene expression induced by nifedipine, ^5^ ^,^ ^8^ phenytoin ^1^ and CsA. ^6^

A question to be raised in this study is whether GO has completed its development under the experimental design as several studies indicated a phased evolution of GO. ^30^ ^–^ ^33^ Studies in rats ^32^ ^,^ ^33^ and cats ^30^ described an initial, but transitory, increase in fibroblasts, followed by the return to previous rates of number of cells in relation to ECM. This fact may justify, at least in part, some contradictory results described previously in studies in which the changes in cell number were assessed in GOs. The aforementioned transitory increase in fibroblast number was not observed in this study, and the authors could not determine whether it occurs in this animal model.

## Conclusions

Within the limits of this study, it can be concluded that CsA, nifedipine and phenytoin do not induce to myofibroblast transdifferentiation in gingival tissue of capuchin monkeys ( *Sapajus spp* ). The methodology also permitted concluding that these drugs do not have a proliferative or antiapoptotic effect on gingival connective cells. The results indicate that the fibrosis induced by the aforementioned drugs in different sites may have specific molecular pathways in a tissue- dependent manner.
